# Demographic and occupational predictors of early response to a mailed invitation to enroll in a longitudinal health study

**DOI:** 10.1186/1471-2288-7-6

**Published:** 2007-01-25

**Authors:** Jean-Paul Chretien, Laura K Chu, Tyler C Smith, Besa Smith, Margaret AK Ryan, the Millennium Cohort Study Team

**Affiliations:** 1 Department of Defense Global Emerging Infections Surveillance and Response System (DoD-GEIS), Walter Reed Army Institute of Research, Silver Spring, MD, USA; 2 Department of Defense Center for Deployment Health Research, at the Naval Health Research Center, San Diego, CA, USA

## Abstract

**Background:**

Often in survey research, subsets of the population invited to complete the survey do not respond in a timely manner and valuable resources are expended in recontact efforts. Various methods of improving response have been offered, such as reducing questionnaire length, offering incentives, and utilizing reminders; however, these methods can be costly. Utilizing characteristics of early responders (refusal or consent) in enrollment and recontact efforts may be a unique and cost-effective approach for improving the quality of epidemiologic research.

**Methods:**

To better understand early responders of any kind, we compared the characteristics of individuals who explicitly refused, consented, or did not respond within 2 months from the start of enrollment into a large cohort study of US military personnel. A multivariate polychotomous logistic regression model was used to estimate the effect of each covariate on the odds of early refusal and on the odds of early consent versus late/non-response, while simultaneously adjusting for all other variables in the model.

**Results:**

From regression analyses, we found many similarities between early refusers and early consenters. Factors associated with both early refusal and early consent included older age, higher education, White race/ethnicity, Reserve/Guard affiliation, and certain information technology and support occupations.

**Conclusion:**

These data suggest that early refusers may differ from late/non-responders, and that certain characteristics are associated with both early refusal and early consent to participate. Structured recruitment efforts that utilize these differences may achieve early response, thereby reducing mail costs and the use of valuable resources in subsequent contact efforts.

## Background

Survey instruments play an important role in epidemiologic research, and due to the relative ease and cost benefits they are frequently implemented utilizing the mail system. Although more convenient than telephone or in-person interviews, postal questionnaires have been found to yield lower response rates [1]. Declining response rates increase the concern that non-participation bias may substantially affect the results of the study. Differences in demographic characteristics between participants and nonparticipants in health survey research are well studied and suggest associations between survey participation and gender [2-4], age [3,5,6], ethnicity [7], and socioeconomic status [4,8]. However, these associations are inconsistent, possibly due to differences in survey methodology, type, or population between studies.

Further studies of non-response have shown that nonparticipants do not necessarily constitute a homogeneous group [9,10]. Since expressed refusal requires the willingness and capacity to register the disinclination to participate, a degree of involvement greater than that required for no reply, it seems plausible that people who explicitly refuse to participate might differ systematically from nonparticipants who simply do not respond. One study investigated three non-response subgroups (refusal, relocation, and illness or death) to an invitation to participate in a cross-sectional survey study of dementia [9]. When compared with participants, a higher proportion of refusers were older, while a higher proportion of those who failed to participate due to relocation were younger. Non-response due to death or illness was associated with male gender and older age.

Little attention has been directed toward early response to postal questionnaires, a noteworthy topic in survey research methodology. With increasing budgetary constraints, studies are charged with developing new strategies to more efficiently use resources while maintaining the integrity of the science. Various methods for improving response, such as offering incentives, and utilizing reminders [11,12], are costly and can be unsuccessful. In the pilot study of the Millennium Cohort Study, response rates were not significantly different between those receiving and not receiving a nominal incentive offered up front, regardless of the type of incentive offered. On the other hand, structured recruitment efforts that utilize differences in subgroups of responders may be a cost-effective method of achieving early response, thereby reducing mail costs and the use of valuable resources in subsequent contact efforts. To better understand early responders of any kind, we sought to compare the characteristics of individuals who refused, consented, or did not respond soon after being mailed an invitation to participate in a large cohort study of US military personnel.

## Methods

### Data Sources

This study was conducted in compliance with all applicable federal regulations governing the protection of human subjects in research (Protocol NHRC.2000.007). Demographic, deployment, and occupational data were obtained from the Defense Manpower Data Center, Seaside, California. Enrollment, refusal, and self-reported data were obtained from the Millennium Cohort Study Team [13], Department of Defense Center for Deployment Health Research, San Diego, California.

### Study Population

In response to the Department of Defense recommendation for a coordinated effort to study the potential effects of deployment-related exposures [14], and bolstered by the Institute of Medicine's recommendation for a systematic, longitudinal, population-based assessment of service members' health [15], the Millennium Cohort Study was launched in October 2000 [13].

Participants were asked to complete one survey (by mail or online) every three years, through 2022, in order to follow potential developments in the health of these participants over a long period of time. The questionnaire included more than 450 questions on general health, personal habits (smoking, alcohol use), occupations, military exposures, and basic demographic and contact information [16]. A total of 256,262 US military personnel were invited to participate in this study by completing a baseline questionnaire. The invited sample was provided by the Defense Manpower Data Center and consisted of randomly chosen participants from the US military over-sampled for Reserve and National Guard personnel, female service members, and those recently deployed to ensure adequate statistical power to detect differences in these smaller subgroups. The probability-based sample represented approximately 11.3% of the 2.2 million men and women in service as of October 1, 2000.

Enrollment in the first panel of the Millennium Cohort Study began in July 2001 and concluded in July 2003. Demographic and occupational data provided by the Defense Manpower Data Center included gender, age, education level, marital status, race/ethnicity (White, American Indian, Asian/Pacific Islander, Black, and Hispanic), service branch (Army, Navy, Marine Corps, Air Force, and Coast Guard), duty status (active duty, Reserve, and National Guard), deployment experience to southwest Asia, Bosnia, or Kosovo in the 2 years prior to October 1, 2000, and Department of Defense primary occupational specialty (10 major groups, defined by the Department of Defense Occupational Conversion Manual [17]). Individuals were excluded from the analysis who were never contacted during the 2-year enrollment due to invalid mailing addresses (n = 38,261) or had missing covariate data (n = 3,610). The remaining 214,391 form the basis of this analysis.

Three groups of study participants were identified based on the date of their response to the Millennium Cohort questionnaire. Early consenters and early refusers were defined as those who submitted a consented questionnaire or explicitly refused to participate prior to September 1, 2001. This date was selected since it was exactly two months after the start of enrollment and just prior to both the September 11 and 2001 anthrax attacks. Increased screening of mail, as well as the closing of several postal facilities soon after the attacks, may have had an affect on the response times of the remaining invitees.

### Statistical analyses

We compared the characteristics of early responders (refusal or consent) and late/non-responders in the Millennium Cohort Study to gain a better understanding of early response. The outcome of interest for all analyses was response to the invitation to participate in the cohort study, categorized as early refusal, early consent, or late/non-response. Univariate analyses, including frequencies and chi-square tests, were used to measure associations of demographic and occupational variables with early refusal or consent to participate. A multivariate polychotomous logistic regression model was used to estimate the effect of each covariate on the odds of early refusal and on the odds of early consent versus late/non-response, while simultaneously adjusting for all other variables in the model. Statistical modeling, producing odds ratios (ORs) and associated 95% confidence intervals (CIs), was performed using SAS software (Version 9.1.3, SAS Institute, Inc., Cary, NC).

## Results

Of the 214,391 US military personnel invited to participate in the Millennium Cohort Study, 704 communicated via e-mail, telephone, or written correspondence their unwillingness to participate prior to 01 September 2001. These individuals represented the early refusal group. The early consenter group consisted of 21,820 participants who completed a paper or online survey prior to 01 September 2001. The remaining 191,867 potential participants were either late refusers (n = 4,092), late responders (n = 55,227) or late non-responders (n = 132,548). Because enrollment efforts of the Millennium Cohort continued into 2003, the group who neither refused nor consented early includes individuals who subsequently refused or consented to participate in the study. Differences between overall responders and nonresponders are described elsewhere [16].

Statistically significant differences among early refusers, early consenters, and late/non-responders were found for all demographic and military characteristics (Table 1). A higher proportion of women were early consenters than early refusers or late/non-responders. Early response (refusal or consent) was associated with age, education, marital status, race/ethnicity, service branch, Reserve/National Guard duty status, and history of past deployment to southwest Asia, Bosnia, or Kosovo. Refusal and consent proportions also varied among the military occupational categories.

**Table 1:** Characteristics of early refusers, early consenters, and late/non-responders for enrollment into the Millennium Cohort Study

	**Early Refuser**^†^		**Early Consenter**^‡^		**Late/Non-responder**^§¶^	
	**N**	**%**	**N**	**%**	**N**	**%**

**Sex**						
Male	537	76.3	15,283	70.0	147,497	76.9
Female	167	23.7	6,537	30.0	44,370	23.1
						
**Age quartile (years)**						
17–23	88	12.5	2,994	13.7	47,596	24.8
24–29	106	15.0	3,761	17.2	46,958	24.5
30–37	178	25.3	6,186	28.4	51,107	26.6
≥38	332	47.2	8,879	40.7	46,206	24.1
						
**Education**						
No high school diploma	22	3.1	1,330	6.1	14,961	7.8
High school graduate	187	26.6	8,436	38.7	96,903	50.5
Some college	211	30.0	5,723	26.2	46,783	24.4
College graduate	147	20.9	3,874	17.7	22,953	12.0
Advanced degree	137	19.4	2,457	11.3	10,267	5.3
						
**Marital status**						
Married	492	69.9	14,074	64.5	103,076	53.7
Not married	212	30.1	7,746	35.5	88,791	46.3
						
**Race/ethnicity**						
White non-Hispanic	543	77.1	16,294	74.7	124,676	65.0
Black non-Hispanic	55	7.8	2,441	11.2	36,713	19.1
Hispanic	31	4.4	1,170	5.4	14,245	7.4
Asian/Pacific Islander	64	9.1	1,474	6.7	11,778	6.2
American Indian	6	0.9	194	0.9	1,731	0.9
Other	5	0.7	247	1.1	2,724	1.4
						
**Service branch**						
Army	201	28.6	9.907	45.4	86,120	44.9
Air Force	326	46.3	6,849	31.4	55,576	29.0
Navy	146	20.7	3,809	17.5	35,313	18.4
Marine Corps	21	3.0	982	4.5	12,635	6.6
Coast Guard	10	1.4	273	1.2	2,223	1.1
						
**Duty status**						
Active duty	313	44.5	9,734	44.6	103,069	53.7
Reserve/National Guard	391	55.5	12,086	55.4	88,798	46.3
						
**Previous deployment experience**^#^						
Non-deployed	502	71.3	15,873	72.7	134,177	69.9
Deployed	202	28.7	5,947	27.3	57,690	30.1
						
**Occupational category**						
Electrical/mechanical	80	11.4	2,923	13.4	31,798	16.6
Combat specialists	169	24.0	4,281	19.6	40,325	21.0
Electronic equipment repair	73	10.4	1,837	8.4	15,388	8.0
Communications/intelligence	39	5.5	1,481	6.8	12,827	6.7
Health care specialists	67	9.5	2,601	11.9	15,794	8.2
Other technical & allied specialists	12	1.7	580	2.7	4,722	2.4
Functional support specialists	158	22.4	4,562	20.9	34,713	18.1
Craft workers	23	3.3	700	3.2	6,852	3.6
Service and supply handlers	48	6.8	1,854	8.5	17,212	9.0
Trainees, other	35	5.0	1,001	4.6	12,236	6.4

** First enrollment into the Millennium Cohort Study.

^† ^Early refusers are invitees who communicated their unwillingness to participate in the study prior to 01 September 2001.

^‡ ^Early consenters are invitees who completed an online or paper survey prior to 01 September 2001.

^§ ^Late/non-responders are invitees who responded after 01 September 2001 or failed to respond in any manner.

^¶ ^Differences between early refusers, early consenters, and late/non-responders were tested with the Pearson chi-square test of association. All *p *values were < 0.001.

^# ^Previous deployment experience to southwest Asia, Bosnia, or Kosovo between 1998 and 2001.

**Figure 1:**
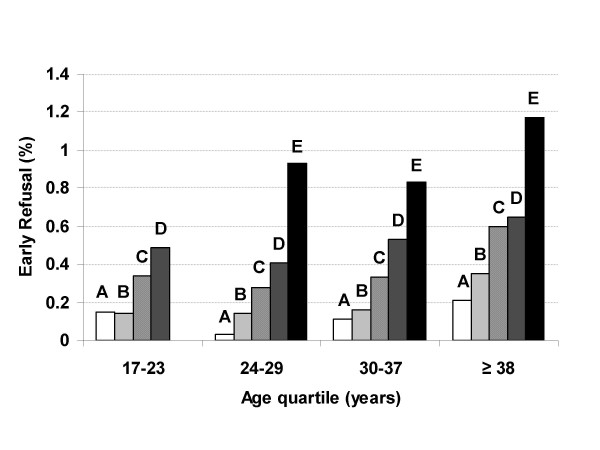
**Percent of invitees refusing early to the Millennium Cohort Study by age and education**. A: No high school diploma; B: High school graduate; C: Some college; D: College graduate; E: Advanced degree

**Figure 2:**
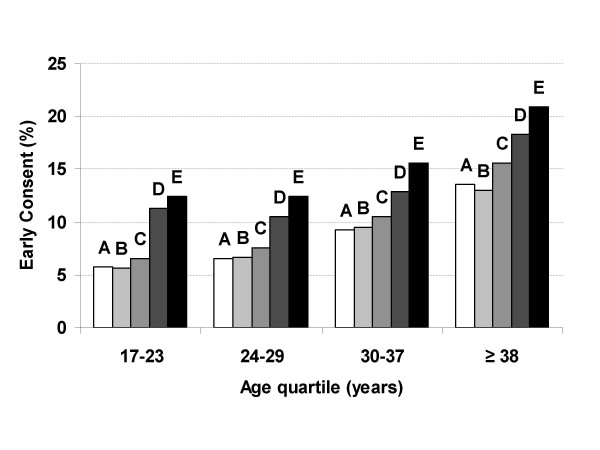
**Percent of invitees consenting early to the Millennium Cohort Study by age and education**. A: No high school diploma; B: High school graduate; C: Some college; D: College graduate; E: Advanced degree

Figure 1 demonstrates the consistency of the association between higher education level and probability of early refusal over strata of age. Individuals with a higher education level were more likely to refuse than those with a lower education level within most age quartiles. Figure 1 also shows an age effect independent of education, with individuals who did not graduate high school, graduated high school but did not attend college, attended college but did not graduate, or graduated college but did not attain an advanced degree and belonged to the oldest age category (= 38 years) more likely to refuse than members of the youngest age category (17–23 years) who achieved the same education level (*p *< 0.001). Figure 2 depicts similar trends for early consent.

The three levels of response for the polychotomous logistic regression analysis were early refusal, early consent, and late/non-response (reference level). The results of this analysis are shown in Table 2. Adjusting for all demographic and military characteristics, the association between women and early consent was statistically significant (OR = 1.62, 95% CI: 1.56, 1.68). The relationship between age and early response is also notable and consistent. An odds ratio of 1.03 (95% CI: 1.02, 1.04) per year implies that a 50-year-old invitee would have 1.90 greater odds of being an early refuser, compared with a 20-year-old invitee. The strongest association found in the multivariate analysis was the relationship between advanced education and early response. Advanced degree status was associated with both early consent and early refusal, but most strongly with the latter (OR = 4.03, 95% CI: 2.48, 6.55).

**Table 2:** Adjusted* polychotomous logistic regression modeling for odds of early refusal and early consent

	**Early Refusal**^† ^**vs. Late/Non-response**^‡^		**Early Consent**^§^** vs. Late/Non-response**^‡^	
	**OR**^¶^	**95% CI**^¶^	**OR**^¶^	**95% CI**^¶^

**Sex**				
Male^#^	--	--	--	--
Female	1.14	(0.94, 1.39)	1.62	(1.56, 1.68)
				
**Age**				
Per year	1.03	(1.02, 1.04)	1.04	(1.04, 1.04)
				
**Education**				
No high school diploma^#^	--	--	--	--
High school graduate	1.25	(0.79, 1.95)	1.08	(1.02, 1.15)
Some college	1.93	(1.21, 3.09)	1.30	(1.21, 1.39)
College graduate	2.42	(1.51, 3.87)	1.51	(1.41, 1.62)
Advanced degree	4.03	(2.48, 6.55)	1.74	(1.61, 1.89)
				
**Marital status**				
Married^#^	--	--	--	--
Not married	0.79	(0.66, 0.94)	0.86	(0.83, 0.89)
				
**Race/ethnicity**				
White non-Hispanic^#^	--	--	--	--
Black non-Hispanic	0.44	(0.33, 0.59)	0.49	(0.47, 0.52)
Hispanic	0.67	(0.46, 0.96)	0.69	(0.64, 0.73)
Asian/Pacific Islander	1.18	(0.89, 1.56)	0.75	(0.71, 0.80)
American Indian	0.85	(0.38, 1.91)	0.87	(0.75, 1.01)
Other	0.54	(0.22, 1.30)	0.72	(0.63, 0.82)
				
**Service branch**				
Army^#^	--	--	--	--
Air Force	1.88	(1.52, 2.32)	0.79	(0.76, 0.82)
Navy	1.96	(1.55, 2.46)	0.92	(0.88, 0.96)
Marine Corps	0.97	(0.61, 1.54)	0.90	(0.84, 0.96)
Coast Guard	1.69	(0.89, 3.23)	0.91	(0.80, 1.03)
				
**Duty status**				
Active duty^#^	--	--	--	--
Reserve/National Guard	1.24	(1.05, 1.46)	1.10	(1.06, 1.13)
				
**Previous deployment status**				
Non-deployed^#^	--	--	--	--
Deployed	0.84	(0.70, 1.01)	1.05	(1.01, 1.09)
				
**Occupational category**				
Electrical/mechanical^#^	--	--	--	--
Combat specialists	1.41	(1.06, 1.87)	1.02	(0.97, 1.07)
Electronic equipment repair	1.45	(1.05, 2.01)	1.13	(1.07, 1.21)
Communications/intelligence	1.13	(0.77, 1.68)	1.13	(1.05, 1.21)
Health care specialists	1.04	(0.73, 1.49)	1.16	(1.09, 1.24)
Other technical & allied specialists	0.98	(0.53, 1.81)	1.20	(1.09, 1.32)
Functional support specialists	1.51	(1.14, 2.01)	1.12	(1.06, 1.18)
Craft workers	1.23	(0.77, 1.96)	1.04	(0.95, 1.14)
Service & supply handlers	1.19	(0.83, 1.73)	1.06	(0.99, 1.12)
Trainees, other	1.78	(1.17, 2.72)	1.05	(0.97, 1.14)

* Adjusted for all variables listed.

^† ^Early refusers are invitees who communicated their unwillingness to participate in the study prior to 01 September 2001.

^‡ ^Late/non-responders are invitees who responded after 01 September 2001 or failed to respond in any manner.

^§ ^Early consenters are invitees who completed an online or paper survey prior to 01 September 2001.

^¶ ^OR = adjusted odds ratio; CI = 95% confidence interval.

^# ^Reference category.

Characteristics significantly associated with both early consent and early refusal included older age, more advanced education, being married, White race/ethnicity, Reserve/Guard status, and occupations in electronic equipment repair and functional support. Characteristics significantly associated with early refusal, but not early consent, include Navy and Air Force affiliation, and the occupational categories of combat specialists and trainees, other. In contrast, characteristics significantly associated with early consent, but not refusal, include female gender, Army affiliation, deployment in the 2 years prior to October 2000, and occupations in communications/intelligence, health care, and other technical and allied specialties.

## Discussion

We used enrollment data from a large cohort study to compare the characteristics of individuals who responded differently to a mailed invitation to participate. Unlike previous studies of early response, we compared both early refusers and early consenters to late/non-responders in an effort to better understand early response of any kind. Certain characteristics were associated with early response as categorized by early refusal and early consent to participate. Multivariate regression analysis revealed that older age, higher education, White race/ethnicity, Reserve/Guard status, and working in electronic equipment repair or functional support occupations were independently and consistently associated with both early refusal and early consent.

Finding common predictors of early refusal and early consent suggest that certain characteristics may influence the probability of early response, whether explicit refusal or consent, as opposed to no response. In our study, individuals who explicitly refused to enroll required the resources to communicate with study investigators through e-mail, telephone, or written correspondence and the opportunity and motivation to use them soon after receiving the invitation. Since subjects who consented to enroll also required these resources, it is not implausible that early refusers and early consenters might share characteristics that determine or reflect the potential to respond in any manner.

Previous studies of non-response have shown higher rates of participation in mailed health surveys for women [2,3,6], older people [5], White race/ethnicity [7], and for people of higher socioeconomic status [4,8], although the associations are not entirely consistent. Demographic differences between early and late consenters have also been reported: subjects who require fewer mailings are more often female [10,18], older [18], more educated [1], and White [7,18]. Furthermore, studies of initial response, consent or refusal, have shown consenters to be younger, more highly educated, and more likely to be White than initial refusers (those who initially refused to participate, but agreed after recontact) [7,19-21]. Psychological and sociological theories have been offered that explain some of these associations. For example, an application of social exchange theory posits that when an institution (such as a government or a business) administers a survey to its members, individuals of higher standing may feel the greatest obligation to contribute back, in the form of participation, to a system from which they have benefited [22]. This could explain the higher participation rates observed in several studies among individuals of higher socioeconomic status [22], and might partially explain why older, more-educated people, and those employed in the health care field consented more promptly in our study.

Characteristics that predict refusal are well studied. Refusers are more frequently women [23], older [7,9,20,21,23-25], non-White [20,21], and of lower educational level [7,20,21,26] than participants. In contrast, we found early refusal to be associated with White race/ethnicity and higher educational level. This difference may be attributable to dissimilarities in study design, as most of these studies were either telephone or in-person surveys, or it may be that certain characteristics of highly educated professional groups predispose them to explicit refusal rather than simple non-participation. For example, people of higher social standing may feel that the risk to their social position of breach of confidentiality outweighs any benefit of participation, or highly educated individuals may become frustrated more easily by multiple-choice questions that they find overly simplistic [27]. While these theories may describe the motivations for non-participation of some of the highly educated individuals who refused in our study, they do not explain readily why this subgroup of nonparticipants chose refusal instead of non-response to express their desire not to participate or why those of White race/ethnicity were more likely to refuse. One possibility is that these individuals simply wished more strongly not to participate. Or, they may have been less timid about registering their refusal to participate with study investigators.

Access advantages may have also prompted early response. The mailed invitations included a paper survey, but also provided a Web address where an online version of the questionnaire could be completed as well as an e-mail address where invitees could request removal from the mailing list. Occupational environments that require computer skills or where email and internet access are more readily available might encourage early response using these methods. The findings from this study suggest this may be true; all occupational categories, except craft workers and service and supply handlers, were more likely to respond early. This is exemplified by significantly higher odds of both early refusal and early consent by personnel working in functional support and administration, and electronic equipment repair. Additionally, those employed in other computer-savvy occupations, such as communications and intelligence and other technical and allied specialties, were significantly more likely to consent early. Although these results suggest that access advantages may have played a role in early response, it does not explain why certain occupational groups chose refusal rather than consent.

The demographic and occupational differences found between early refusers and individuals who neither refused nor consented early may have implications for survey research methodology and epidemiologic enrollment efforts. If the characteristics that distinguish early refusers from other nonparticipants are associated with the variables under investigation, then standard methods of correction for non-participation could benefit from consideration of the heterogeneity among subgroups of nonparticipants. For example, one approach to reduce non-participation bias is to use information from a sample of nonparticipants in the statistical adjustment of results for the participants [28]. This method might provide more precise estimates of parameters in the target population with stratification by mode of non-participation, perhaps by sampling early refusers separately from other nonparticipants.

If a subgroup of the target population is especially likely to refuse enrollment soon after being invited, the identification of this subgroup could allow costly efforts to recruit non-respondents to be targeted toward people who are ultimately more likely to enroll. In our study, many early refusers used the option of declining enrollment through e-mail. Although this required greater effort than simply ignoring the invitation, it was an easy method of refusal for some individuals who might have been less likely to refuse explicitly had refusal required written correspondence. In that case, these subjects might have accounted for an especially low-yield target for subsequent mailings. Besides reducing the cost of future mailings, eliminating early refusers from mailing lists might also prevent these individuals from feeling anger or frustration at receiving additional invitations to participate. This could reduce the chance that they would engage in organized anti-survey activity in the future [27]. The available data suggest that providing the option of explicit refusal in a mail survey may increase the rate of explicit refusal without increasing overall non-participation [29].

Although the percentage of refusers in this study was approximately 0.3%, continued contact with those who do not intend to participate can be costly, both financially and in terms of response, even if the subset is small. If, for example, repeated mailings and e-mail reminders anger refusers to the point of spreading negative press, potential responders may be swayed into nonparticipation, increasing the potential for bias. Furthermore, if participants were to refuse or consent early – that is, after the first invitation – the high monetary cost of each cycle of mailed invitations and surveys could be reallocated toward other areas of the study such as retainment.

Several limitations of this study should be considered when interpreting the results. It is possible that some of the differences in early refusal and early consent rates among subgroups of the target population are explained by differences in rates of receipt of the invitation to enroll. In this military population, younger and less-educated people may have been less likely to receive the invitation because of more frequent duty station reassignments or deployments, or lack of access to e-mail. In addition, the study population used in this investigation is a subset of military personnel and may not be representative of the US military as a whole or the general population. The US military is comprised predominantly of men and is more educated, younger, and ethnically diverse than the general US population [30]. These differences may help to explain some of the dissimilarities in characteristics of early refusers and consenters encountered between the present study and previously published work. However, there is no evidence to suggest that members of the military have a systematically different approach to answering requests for participation, as long as participation is voluntary. Regardless, the results of this study should be interpreted cautiously, as they may be conceptually relevant to studies in other populations, but may not have similar predictors.

Although we have focused on early refusal and consent, the characteristics associated with these events may not be associated with ultimate refusal or consent. If late consenters are more similar to non-responders than early consenters are, a later comparison might show greater similarity among consenters and non-responders and greater difference between consenters and early refusers. However, the goal of our investigation has not been to identify characteristics associated with eventual consent or terminal non-response, a topic that has received much attention. Rather, the finding that early refusers share certain characteristics with early consenters, which distinguish them from those who do not respond early, suggests that this subgroup of nonparticipants may deserve special consideration in study design and analysis.

The existence of a demographically distinct group of early refusers would be less relevant to methods of response bias correction if the ultimate health outcomes under investigation were not associated with demographic characteristics. We cannot assess whether early refusers might be more or less likely to develop outcomes of interest than those who neither refused nor consented early. It may be that this question can only reliably be answered retrospectively for certain outcomes, since refusers may be at greater risk than consenters for unfavorable health outcomes, even if they are similar at baseline in demographic characteristics and general measures of health [31].

A strength of this study is the availability of demographic and occupational data on all members of the invited population, regardless of participation in the study. Some studies of non-participation rely on follow-up interviews or questionnaires on a sample of nonparticipants to characterize the entire group, but the proportion of initial non-respondents who complete a follow-up questionnaire may be quite low [32,33]. Follow-up interviews can be time-consuming or costly, and might only be conducted on a subset of non-respondents [2]. Additionally, this study has a large and diverse study population, which allowed for robust comparisons between early and late/non-responders and greater generalizability than in previous studies of response.

## Conclusion

We identified demographic and occupational similarities among early refusers and early consenters which distinguish both groups from individuals who did not respond promptly to a mailed invitation to enroll in a large cohort study. Early refusers may constitute a distinct group of nonparticipants who have the desire and opportunity to communicate their wish not to enroll. Consideration of the potential heterogeneity among subgroups of nonparticipants in recruitment efforts could reduce the overall cost of enrollment while improving the quality of survey-based health studies.

## Competing interests

The author(s) declare that they have no competing interests.

## Authors' contributions

JPC, TCS, BS, and MAKR contributed to all aspects of this publication, including design, statistical analyses, interpretation, and drafting of the manuscript. LKC assisted in statistical analyses and drafting of the manuscript. All authors read and approved the final manuscript.

## Pre-publication history

The pre-publication history for this paper can be accessed here:


